# Developing a Noninvasive Procedure Using Labeled Monoclonal Antibody Anti-VEGF (Bevacizumab) for Detection of Endometriosis

**DOI:** 10.1155/2015/751460

**Published:** 2015-07-09

**Authors:** Daniel Escorsim Machado, Jamila Alessandra Perini, Margarida Maria Camoes Orlando, Ralph Santos-Oliveira

**Affiliations:** ^1^Laboratório de Pesquisa de Ciências Farmacêuticas, Unidade de Farmácia, Centro Universitário Estadual da Zona Oeste, Rio de Janeiro, RJ, Brazil; ^2^Programa de Pós-Graduação em Saúde Pública e Meio Ambiente, Escola Nacional de Saúde Pública, Fundação Oswaldo Cruz, Rio de Janeiro, RJ, Brazil; ^3^University Hospital Pedro Ernesto, Nuclear Medicine Service, Rio de Janeiro, RJ, Brazil; ^4^Laboratory of Nanoradiopharmaceuticals, 21741706 Rio de Janeiro, RJ, Brazil

## Abstract

The off-label use of bevacizumab labeled with 99mTc as a new radiopharmaceutical for imaging of endometriosis is a promising noninvasive, new clinical procedure. The bevacizumab in monoclonal antibodies targeted at vascular endothelial growth factor (VEGF) is superexpressed in cases of endometriosis. In this study we evaluate the imaging of endometriosis lesion in rats (induced to endometriosis) using bevacizumab-99mTc. The results showed that bevacizumab-99mTc imaged the lesion and support his use for Nuclear Medicine applied to gynecology. Also the results appointed that this radiopharmaceutical has a hepatobiliary excretion. It is important to notice that the dose used was almost 0,01% of the usual dose for the bevacizumab.

## 1. Introduction

Endometriosis is a gynecological disease defined as the presence of growth functional endometrium outside the uterus [1]. This disease is associated with infertility and incapacitating painful symptoms, including chronic pelvic pain, dysmenorrhea, and dyspareunia [2]. It is important to notice that approximately 11% of women did not report any gynecologic pain symptoms [3]. It is a benign disease but it can behave like a malignant one in terms of growing, infiltrating, and adhering to the surrounding tissues [4]. However, the pathogenesis of endometriosis has not yet been fully clarified. The most widely accepted theory for the development of endometriosis is the implantation theory of Sampson [5], who proposed that endometrial tissue is shed in a retrograde manner into the peritoneal cavity during menstruation, where it attaches and proliferates at ectopic sites. In addition to the retrograde flow of exfoliated endometrium, new blood vessels essential for the survival of the endometrial implant, and therefore the development of endometriosis, must be formed [6].

The pathological angiogenesis occurs in a range of diseases that could be classed together as angiogenesis-dependent diseases, and the endometriosis has been assigned to this group [7]. Endometrial angiogenesis is promoted by numerous inducers and growth factors, including vascular endothelial growth factor (VEGF). Several authors postulated that VEGF-induced angiogenesis is a critical aspect of the pathophysiology of this disease [8–11].

Moreover, the endometriosis is regarded as a complex and heterogeneous disease, which is associated with diagnosis delay and high recurrence rates. According to the American Congress of Obstetricians and Gynecologists, the only way to definitively diagnose endometriosis (of any type) is direct visualization of the endometriotic implant by a trained surgeon followed by biopsy and histological confirmation as the gold standard; in any case an invasive procedure is required [12]. As angiogenesis represents a critical step in the establishment and pathogenesis of endometriosis; this process has been viewed as a potential new target for better diagnoses of disease.

Bevacizumab is a recombinant humanized monoclonal antibody IgG1 which selectively binds to and neutralizes the biological activity of vascular endothelial growth factor (VEGF) and inhibits its interaction with receptors VEGFR 1 and VEGFR 2 [13]. Bevacizumab monoclonal antibody was approved for treatment of first and second line metastatic colorectal cancer; in combination with 5-fluorouracil, it is also approved for the treatment of first line non-small cell lung cancer and for unresectable, locally advanced, recurrent, or metastatic disease [1] as well as metastatic breast cancer when combined with carboplatin and paclitaxel. It was recently approved (November 2014) for platinum-resistant recurrent epithelial ovarian cancer in combination with chemotherapy (paclitaxel, pegylated liposomal doxorubicin, or topotecan). There is also evidence of the effectiveness of bevacizumab as the sole medication in metastatic clear cell renal cancer [13, 14] or combined with erlotinib [14, 15], but it was only approved for the treatment of renal cell carcinoma with interferon-*α* [16]. Finally, bevacizumab has shown antitumour activity in glioblastoma. Based on the improvement in objective response rate, it was approved as a single agent in adult patients with progressive disease following prior therapy because glioblastoma is a highly vascularised tumour with high levels of VEGF [16, 17].

Is important to notice that there is no study of labeling bevacizumab with 99mTc for endometriosis imaging. A good and initial expectation for the use of antiangiogenic agents is that they would be practically free of adverse reactions since the dose for Nuclear Medicine procedure is almost 0,01% of the normal dose used in clinics. The aim of this study was to evaluate the bevacizumab labelled with 99mTct as a new, noninvasive radiopharmaceutical for diagnosis of endometriosis.

## 2. Methodology

### 2.1. Surgical Induction of Endometriosis

The endometriosis model was established as previously described elsewhere [10–12, 18]. In brief, 20 female rats were opened at the abdomen through a 3 cm midline incision to expose the uterus. One uterine horn was ligated at both the uterotubal junction and the cervical end and was removed. The segment was placed in phosphate-buffered saline at 37°C and split longitudinally, and 5 × 5 mm pieces were sectioned. These explants were then anchored onto the peritoneum on the right side of the ventral abdominal wall by nonabsorbable polypropylene sutures (Prolene 6-0, Ethicon, Piscataway, NJ). The abdomen was closed and the animals were allowed to recover from anesthesia. After 15 days, the bevacizumab labeled with 99mTc was administered intraocularly, and the animals were euthanized to collect the endometriotic lesions, blood, and the other organs (spleen, heart, kidney, lung, stomach, brain, liver, and intestine).

All experiments were conducted in accordance with the ethical guidelines from the Institutional Animal Care Committee (CEUA) in Universidade Estadual da Zona Oeste (UEZO) of Rio de Janeiro, Brazil, protocol code: CEUA-UEZO.009/14.

### 2.2. Labeling with 99mTc and Imaging

The labeling process was done using 150 *μ*L of the bevacizumab incubated with stannous chloride (SnCl_2_) solutions (80 *μ*L/mL) (Sigma-Aldrich) for 20 minutes at room temperature. Then this solution was incubated with 100 *μ*Ci (approximately 300 *μ*L) of technetium-99m for another 10 minutes in order to label bevacizumab with 99mTc [19, 20].

#### 2.2.1. Quality Control of the Labeling Process

The quality control of the labeling process was done by TLC (Thin layer Chromatography).

#### 2.2.2. Biodistribution

**Figure 1:**
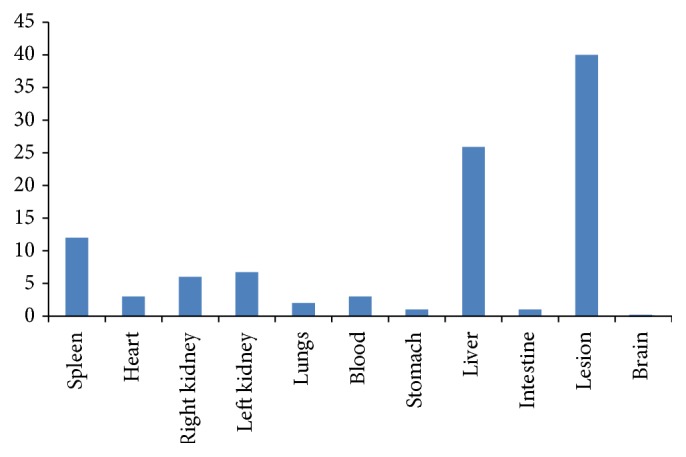
Biodistribution of bevacizumab labeled with 99mTc after 1 hour injection. This is the mean of 15 rats.

The biodistribution studies were done with 15 rats (Wistar) (Figure 1). The final averages were used to construct the graphics. The Institutional Review Board and the Animal Ethics Committee approved the study protocol. The labeled samples (3.7 MBq/0.2 mL) were administered by intraocular injection. Counts were acquired for 5 min in a 15% window centered at 140 KeV after 1 h of the injection. The animals were sacrificed and their organs removed and weighed, and the radioactivity uptake is counted in a gamma counter (Perkin Elmer). Results were expressed as a percentage of injected dose (activity) per gram of tissue [21].

#### 2.2.3. Imaging

**Figure 2:**
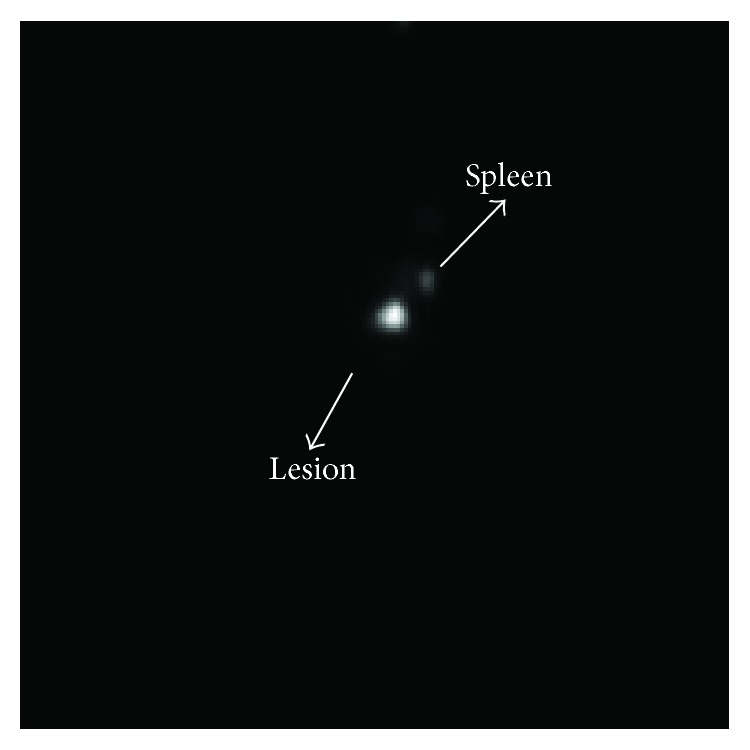
Planar image of the lesion after 1 hour injection.

Planar images (Figure 2) were obtained at 60 min after injection using a Millennium Gamma Camera (GE Healthcare, Cleveland, USA). Counts were acquired for 5 min in a 15% window centered at 140 KeV. The images were processed using* OsiriX *software, and regions of interest (ROIs) over the lesion were selected for specific analysis (Figure 1). The Institutional Review Board and the Animal Ethics Committee of the Federal University of Pernambuco (number 23076.002362/2010-37) approved all studies.

## 3. Results and Discussion

The labeling process showed >99% of the bevacizumab with 99mTc in interval of 24 hours. This supports the labeling process using the direct method.

The results of biodistribution showed a high uptake of bevacizumab-99mTc by the liver and spleen, even in the presence of the lesion (endometriosis). The uptake by the liver suggests fast metabolism of the drug, which is of great interest for a diagnostic drug. The presence in the spleen, lung, and intestine is explained by the fact that the mesenchyme of the intestine and also spleen and lung of rats secretes several ligands for epidermal growth factor that induces differentiation of the epithelial cell sites [8].

The image showed that the lesion was well defined and supports the use of this radiopharmaceutical for endometriosis diagnosis.

The objective of this study was to determine whether labeled monoclonal antibody anti-VEGF (bevacizumab) could be useful in the diagnosis of endometriosis. A study [9, 10] has shown that in endometriotic implants there are a high level of neovascularization, and in consequence a high level of VEGF. Unfortunately, the mean latency of 6-7 years from onset of symptoms to definitive diagnosis of endometriosis may have significant consequences in terms of disease progression. In this direction a noninvasive diagnostic tool could be used more readily by clinicians, perhaps leading to decreased time until diagnosis and subsequently decreased consequences due to disease progression [22]. In addition, the laparoscopy, the invasive procedure surgery, remains the gold standard approach to diagnostic endometriosis and the lack of a laboratory biomarker for the disease remains a top research priority. The discovery of a sufficiently sensitive and specific clinical agent for the nonsurgical detection of endometriosis promises earlier diagnosis in order to provide additional support for treatment planning. Also the development of new radiopharmaceutical that could be used daily in the clinic routine is quite promising.

## 4. Conclusion

The results showed that bevacizumab labeled with 99mTc may be supported to be used as radiopharmaceuticals for endometriosis imaging, instead of surgical procedure. This study is just a preliminary one and further studies are required, especially a cost-effective one, in order to show the real advantages in terms of cost of the use of bevacizumab-99mTc since the monoclonal antibody is still expensive.
